# Characterization and comparison of the bacterial community between complete intensive and extensive feeding patterns in pigs

**DOI:** 10.1186/s13568-021-01191-y

**Published:** 2021-02-25

**Authors:** Xin-Jian Li, Mingyu Wang, Yahui Xue, Dongdong Duan, Cong Li, Jianwei Ye, Xuelei Han, Ruimin Qiao, Kejun Wang, Xiu-Ling Li

**Affiliations:** College of Animal Science and Technology, Henan Agricultural University, Zhengzhou, 450046 China

**Keywords:** 16S rRNA, Feeding patterns, Gut microbiome, Microbial diversity, Pig

## Abstract

To investigate and compare the gut microbiota structures in complete intensive feeding pattern (CP) and extensive feeding pattern (EP) groups, a total of 20 pigs were divided into two groups and fed the same diet. The fecal microbial composition was profiled using 16S rRNA gene sequencing. Our results showed that seventeen predominant genera were present in each pig sample and constituted the phylogenetic core of the microbiota at the class level. The abundance of most of the core microbial flora were significantly higher in the CP group than in the EP group (*P* < 0.05), while the abundance of *Gammaproteobacteria* was significantly lower in the CP group than in the EP group (*P* < 0.05). The CP group had significantly greater community diversity, richness, and evenness than the EP group (*P* < 0.05). Functional prediction analysis indicated that intestinal microbial species potentially led to faster growth and an increased fat accumulation capacity in the CP group; however, disease resistance was weaker in the CP group than in the EP group. In conclusion, EP pigs have a wider range of activity and better animal welfare than CP pigs, which helps reduce the occurrence of diseases and neurological symptoms. To explore the effect of intestinal flora on disease resistance in pigs at the molecular level, *Coprococcus*, which is a key gut bacterium in the intestine, was selected for isolation and purification and cocultured with intestinal epithelial cells. qPCR was performed to determine the effect of *Coprococcus* on SLA-DRB gene expression in intestinal epithelial cells. The results showed that *Coprococcus* enhanced SLA-DRB gene expression in intestinal epithelial cells. The results provide useful reference data for further study on the relationship between intestinal flora and pig disease resistance.

## Introduction

Pigs were domesticated from wild boar in multiple locations across Eurasia approximately 10,000 years ago (Larson et al. 2005) and provide more dietary protein in the human diet than any other animal. Since the mid-20th century, the complete intensive feeding pattern (CP) has become dominant in pig production systems in indoor facilities and on farms. One of the main reasons for the predominance of the CP is that pigs housed in intensive systems can be reared to slaughter-age quickly by feeding the animals a high-protein diet and limiting their activity, also saving on long-term feed costs. However, CP pigs are raised in much smaller spaces, resulting in poor air quality. High concentrations of noxious gases (for example, ammonia) and dust can reduce their ability to resist diseases, including enzootic pneumonia, porcine reproductive and respiratory syndrome (PRRS), swine influenza, and other respiratory problems (Cambra-López et al. 2010; Radon et al. 2002). In contrast, the extensive feeding pattern (EP) is an alternative production system in which pigs are maintained under free-range conditions for part or all of the production cycle. Each pig has enough space to actively move, reducing the occurrence of respiratory and enteric diseases (Carrasco-Garcia et al. 2016; Relun et al. 2015). Pigs produced under outdoor conditions have different meat characteristics, mainly due to exercise or pasture intake, which may affect pH, fat deposition, fatty acid profiles, and meat color. EP pigs may have more comfortable living conditions and experience less environmental stress than CP pigs. This type of rearing affects animal health and the serum biochemical profile. The EP also has an effect on cortisol levels, which are related to immune function, thus improving the disease resistance in EP pigs (Temple et al. 2011). Therefore, in many parts of the world, the EP is still one of the main breeding patterns.

Pigs harbor a large and complex intestinal microbiota, which has a certain impact on pig performance. Some studies have found that feeding piglets calcium-phosphorus can effectively promote the proliferation of intestinal lactobacilli, and the proliferation of lactic acid bacteria can competitively inhibit the growth of potentially pathogenic bacteria and enhance piglet disease resistance (Heyer et al. 2015; Kim and Isaacson 2017) showed that native microorganisms in the intestinal tracts of pigs could exert competitive inhibitory effects on foreign pathogenic bacteria, thus enhancing disease resistance in the host. Different compositions of intestinal microbiota in piglets may affect piglets’ growth performance and absorption capacity (Yang et al. 2018). In addition, many studies have shown that the intestinal flora plays a role in regulating intestinal movement and secretion, decomposing macromolecular complex polysaccharides in food, digesting and absorbing nutrients, maintaining the integrity of the intestinal epithelial barrier, and promoting and maintaining the normal development and activity of the immune system (Clemente et al. 2012; Dinan et al. 2017a).

However, there are few studies on the effects of feeding mode on the diversity of the intestinal flora in pigs. The effects of different feeding methods on animals’ phenotypic traits have been well studied; however, relatively few studies have explored the relationship between the composition of the intestinal microbiota and its phenotypic traits under different feeding modes. Hence, the aim of this study was (1) to explore and compare the fecal bacterial community structures in CP and EP pigs and (2) to discover how the bacterial community may be related to pig performance by functional prediction analysis.

## Materials and methods

### Animals and sample collection

The experimental animals used in this experiment belonged to a hybrid pig breed bred by Chuying Agro-pastoral Group Co., Ltd. Chuying black pigs have the characteristics of good meat quality and coarse feeding resistance. A total of twenty 10-month-old Chuying black pigs, provided by Chuying Agro-pastoral Group Co., Ltd., Henan Province, China, were divided into the CP group (n = 10) and the EP group (n = 10). The feeding trial ran concurrently for the two groups from May to December 2017. During the feeding trial, all pigs were provided the same pelleted feed and received standard vaccinations and medications on a nucleus farm containing 4 rooms. Each group had access to one single-space feeder equipped with feed intake recording equipment (FIRE) (Osborne Industries, Inc., Osborne, KS, USA). Pig identification and feed consumption were recorded every time a pig visited the feeder. In this experiment, the feed intake for the two groups of pigs was strictly controlled to eliminate its influence on the test results. However, the pigs in the EP group were provided rotational grazing and grazing modes, and the grazing time was adjusted according to the weather and feed crops to simulate outdoor activity and freely feeding in the natural environment. To reduce the influence of the pig microbial composition, all animals in the present study had the same genetic background. In this study, the induced defecation method was used to induce defecation in experimental animals. After collecting feces with clean and pollution-free disposable gloves, the feces were loaded into a 50 mL frozen storage tube, immediately placed in a liquid nitrogen container and stored at −80°C.

### DNA extraction and 16S rRNA gene sequencing

DNA extraction from the interior of the frozen fecal samples (250 mg) was performed using MO BIO’s PowerFecalTM DNA Isolation Kit (MO BIO Laboratories, Carlsbad, CA, USA) according to the manufacturer’s protocol. The DNA concentration was measured by a NanoDrop Spectrophotometer. 16S rRNA gene sequencing was performed by the Beijing Genomics Institute (BGI, China) via an Illumina HiSeq 2500 instrument. In brief, the V4 hypervariable regions of the bacterial 16S rRNA gene were amplified using barcoded primers (forward: GTGCCAGCMGCCGCGGTAA, reverse: GGACTACHVGGGTWTCTAAT). Each 30-mL PCR sample contained 30 ng of DNA, 4 µL of PCR Primer Cocktail with 25 µL of PCR Master Mix, and molecular biology-grade water as needed. The PCR conditions were as follows: predenaturation at 98 ℃ for 3 min; followed by 30 cycles of 98 ℃ for 45 s, 55 ℃ for 45 s, and 72 ℃ for 45 s; and final extension at 72 ℃ for 7 min. The overhanging ends of the PCR product were converted into blunt ends using T4 DNA polymerase, Klenow fragment, and T4 polynucleotide kinase. Then, an ‘A’ base was added to each 3’ end to increase the ease of adding adapters. The PCR products were purified with Agencourt Ampure XP beads (Beckman Coulter). After purification, the PCR products were used for the construction of libraries; then, 20 samples were sequenced using a 250-bp paired-end Illumina HiSeq/MiSeq platform (Illumina, CA, USA) at BGI Shenzhen.

### Sequence filtering and taxonomic assignment

To obtain accurate and reliable results, raw reads with an average PHRED score lower than 20 over a 30-bp sliding window with more than a 3 base mismatches in the 15-bp overlap region between the adapter and read, ambiguous bases, homopolymer runs exceeding 10 bp and sequence lengths shorter than 100 bp were filtered out (Fadrosh et al. 2014). Then, clean paired-end reads that overlapped were merged into tags using the FLASH tool (Magoč et al. 2011). The tags were clustered into operational taxonomic units (OTUs) USEARCH scripts (Edgar et al. 2010). In brief, all tags from each sample were initially clustered into OTUs with 97% identity by UPARSE software (Edgar et al. 2013), and unique OTU representative sequences were obtained. Second, the UCHIME algorithm (Edgar et al. 2011) was used to scan for chimeras. Finally, a representative OTU table was generated using ‘usearch_global’ in USEARCH (Edgar et al. 2010) and then classified using the Ribosomal Database Project (RDP) naïve Bayesian classifier v.2.2 (Cole et al. 2014) against the Greengenes 16S rRNA gene database (DeSantis et al. 2006), with 0.6 confidence values. A Venn diagram plot was drawn using the *VennDiagram* package in R (Chen 2011) to compare the OTUs between the EP and CP groups. Principal component analysis (PCA) was also performed to assess bacterial composition and determine microbial community differences among the samples with the ade4 package in R (Dray et al. 2007).

### Statistical analysis

In addition to the species composition and abundance analyses described above, phylogenetic tree construction further clarified the species evolutionary relationships. The representative sequences were aligned against the Silva core set (Silva_108_core_aligned_seqs) by ‘align_seqs.py’ using the PyNAST tool (Caporaso et al. 2010). A representative OTU phylogenetic tree was constructed using built-in QIIME scripts, including the fast tree method for tree construction (Kuczynski et al. 2012). The tags with the highest abundance for each genus were chosen as corresponding representative genus sequences, and the genus-level phylogenetic tree was obtained in the same way as the OTU phylogenetic tree. The phylogenetic trees were imaged by R software (v3.1.1).

The gut microbial richness and diversity indices (observed species, Chao 1 estimator, abundance-based coverage estimation (ACE), Shannon and Simpson indices) were calculated using mothur (Schloss et al. 2009). The Wilcoxon rank-sum test was used to test the differences in α-diversity between the CP and EP groups. A *P* < 0.05 was considered statistically significant.

Phylogenetic measurements of β-diversity were also estimated using QIIME software (Kuczynski et al. 2012). The Bray-Curtis distance, weighted UniFrac distance, and unweighted UniFrac distance were used for principal coordinate analysis (PCoA) to compare the microbial communities in the two groups. Linear discriminant analysis effect size (LefSe) (Segata et al. 2011), which takes into account both statistical significance and biological relevance, was conducted to identify OTUs that were differentially represented between CP and EP Chuying black pigs. PICRUSt (Langille et al. 2013) was utilized to infer functional pathways from the 16S rRNA gene sequencing data and the Greengenes database. Significant differences between pairs of samples or multiple groups of KEGG pathways were visualized using STAMP software (Parks et al. 2014).

### Cell culture and isolation and purification of bacteria

*Coprococcus* were isolated from fecal samples according to the methods of Mirlohi et al. (2009). Then, *Coprococcus* were cultured, and the bacterial solutions with concentration gradients of 10^6^, 10^7^and 10^8^ were prepared and stored at 4 °C. Pig intestinal epithelial cells were isolated and cultured according to the method of Deguchi et al. (2006). After cell culture for 24 h, the cells were treated with the different concentrations of the bacterial solutions prepared in the previous stage. After the cells were cocultured with the bacterial solution for 12 h and collected, RNA was extracted according to the instructions of the RNA extraction kit. The concentration and quantity of RNA were measured using a NanoDrop 1000 Spectrophotometer (NanoDrop, Germany), and then the qualified RNA samples were stored in a refrigerator at a low temperature for later use. A number of studies have shown that there is a significant correlation between the expression of the SLA-DRB gene and disease resistance, so the SLA-DRB gene is considered an important candidate gene for regulating disease resistance (Huang et al. 2016). Based on the above finding, the SLA-DRB gene was selected as the candidate gene in this study to study the influence of intestinal flora on its expression. A Takara kit was used for reverse transcription and qPCR to detect the expression level of the SLA-DRB gene. CFX_3StepAmp + Melt was used for qPCR. HMBS was selected as the internal reference gene.

## Results

### Microbiome sequencing statistics

After removing low-quality reads, an average of 18.71 Mb of clean data remained for each animal. A total of 766,524 16S rRNA high-quality paired-end reads with an average of 38,362 sequences per sample were obtained. The reads were then combined into tags based on their overlap; 764,186 tags were generated in total, with an average of 38,209 tags per sample, and the average length was 252 bp. Detailed information is listed in Additional file 1:  Table S1.

### Pyrosequencing data and microbial diversity

**Fig. 1:**
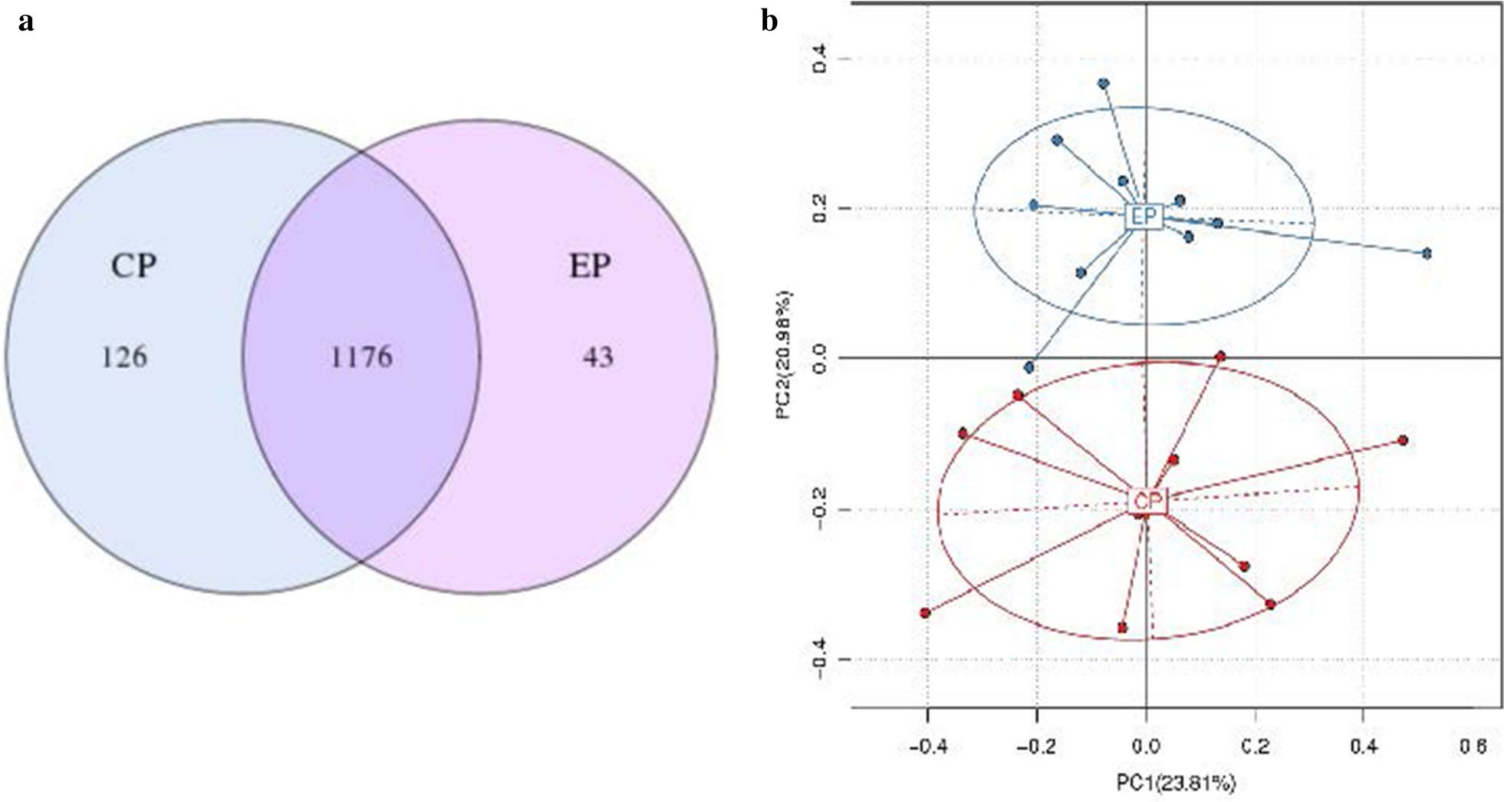
Cluster analysis of the OTUs in CP and EP pigs. **a** Venn diagram showing shared and unique genera in the microbial communities in both groups. The OTUs were defined at a 97% sequence similarity level. **b** PC analysis of the OTUs in the CP and EP groups

A total of 15,732 OTUs (CP = 8222 and EP = 7510) were identified at a 97% sequence similarity level in this study. There were 1176 OTUs shared between the two groups, and the numbers of unique genera in the CP and EP groups were 126 and 43, respectively (Fig. 1a). Furthermore, PCA revealed that the CP and EP groups formed a relatively isolated cluster, while the second PC (accounting for 20.98%) clearly divided these pigs into two groups (Fig. 1b). The microbial communities from the two feeding pattern groups were estimated (Table 1). CP pigs had significantly higher Chao1 and ACE indices than EP pigs, which meant that the CP increased the diversity of the microbial community. However, there were no significant differences in either the Shannon or Simpson indices between the CP and EP groups.

**Table 1:** Estimated OTU richness and diversity indices in CP and EP pigs

Feeding patterns	Chao1	ACE	Shannon	Simpson
CP	938 ± 67^a^	935 ± 65^a^	5.00 ± 0.23	0.019 ± 0.007
EP	847 ± 92^b^	840 ± 95^b^	4.92 ± 0.44	0.020 ± 0.008

Data are presented as the mean ± SD of 10 replicates; different letters in the same row indicate a significant difference (P < 0.05)

### Comparison of the cecal microbial community compositions

**Fig. 2:**
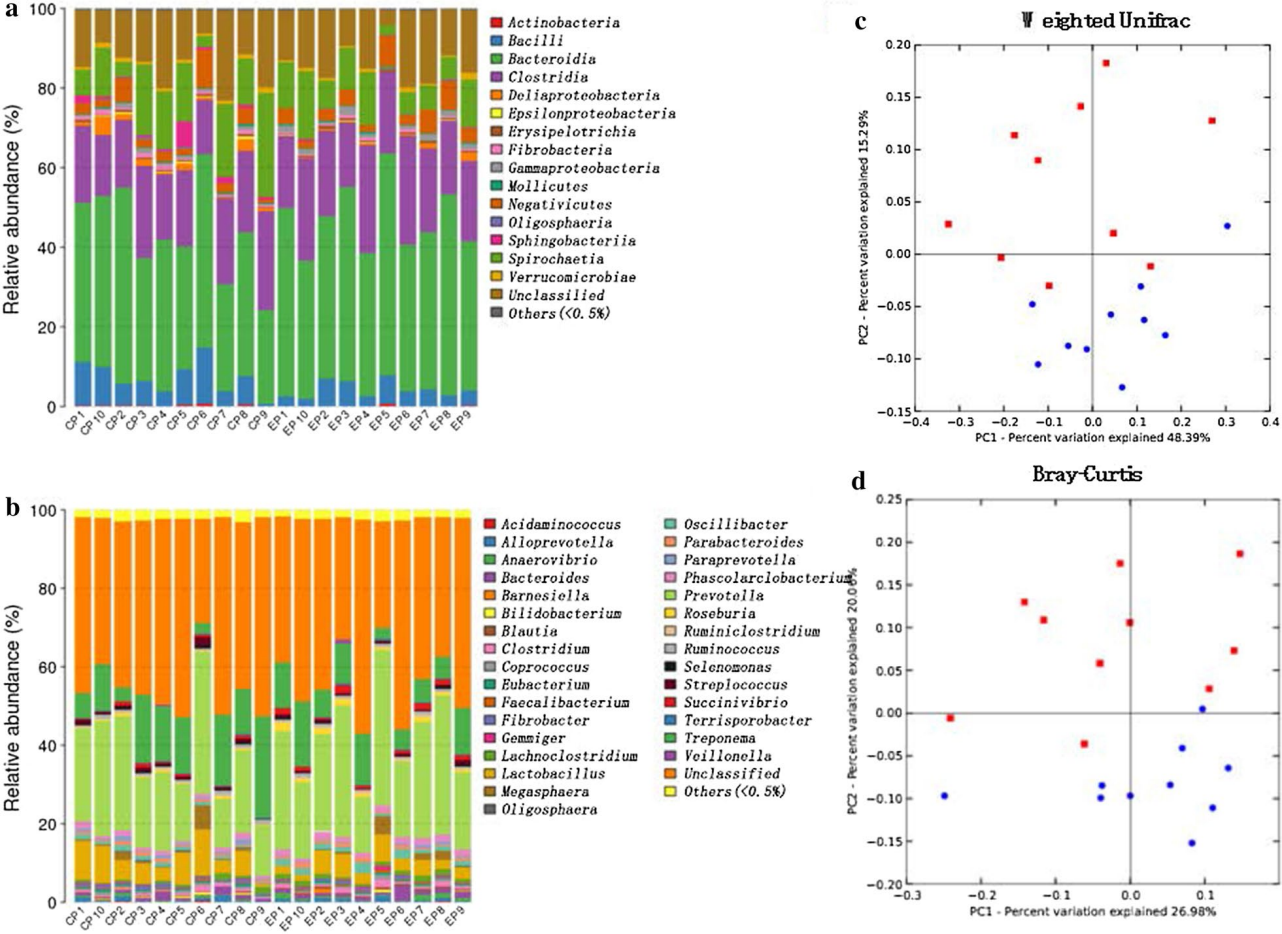
Microbial community compositions in the intestines in the CP and EP groups. Taxonomic composition at the class (**a**) and genus (**b**) levels. Only classes and genera with an abundance > 0.5% in any group were plotted. PCoA of microbiota based on weighted UniFrac (**c**) and Bray-Curtis (**d**) distances. Red dots represent the distribution of CP group samples, while blue dots represent the distribution of EP group samples

A total of 15 bacteria, accounting for more than 95% of all gut microbes at the class level, were classified based on the identified OTUs. Bacteria belonging to the class *Bacteroidia* (36.7% and 42.8%, respectively) were the predominant microorganisms in the CP and EP pigs, followed by *Clostridia* (19.1% and 21.6%, respectively), *Spirochaetia* (12.8% and 9.1%, respectively) and *Bacilli* (7.1% and 4.2%, respectively) (Fig. 2a). Among the core flora, the levels of *Actinobacteria*, *Bacilli*, *Deltaproteobacteria*, *Fibrobacteria*, *Epsilonproteobacteria*, *Oligosphaeria*, *Sphingobacteriia*, and *Spirochaetia* were significantly higher in the CP group than in the EP group (P < 0.05), while *Gammaproteobacteria* was significantly lower in the CP group than in the EP group (P < 0.05). *Bacteroidia*, *Erysipelotrichia*, and *Negativicutes* were also lower in the CP group than in the EP group, although this difference was not statistically significant. The levels of *Coprococcus, Lachnospiraceae*, *Succinivibrio*, *Aeromonadales*, *Roseburia*, *Anaerovibrio*, *Faecalibacterium*, and *Oscilibacter* were significantly higher in the EP group than in the CP group (P < 0.05). At the genus level, bacteria from the genus *Prevotella* were the most prevalent in CP (21.7%) and EP (26.5%) pigs, followed by *Treponema* (12.5% and 8.9%, respectively) and *Lactobacillus* (6.4% and 3.7%, respectively) (Fig. 2b and Additional file 2: Table S2).

Weighted UniFrac distance analysis showed that the community composition and structure in the CP group were significantly different from those in the EP group according to the first two PCs, explaining 48.39% and 15.29% of the variation (Fig. 2c), respectively. Similarly, the taxonomic composition associated with CP pigs was also distantly separated from that associated with EP pigs when the Bray-Curtis distance was used (Fig. 2d).

**Fig. 3:**
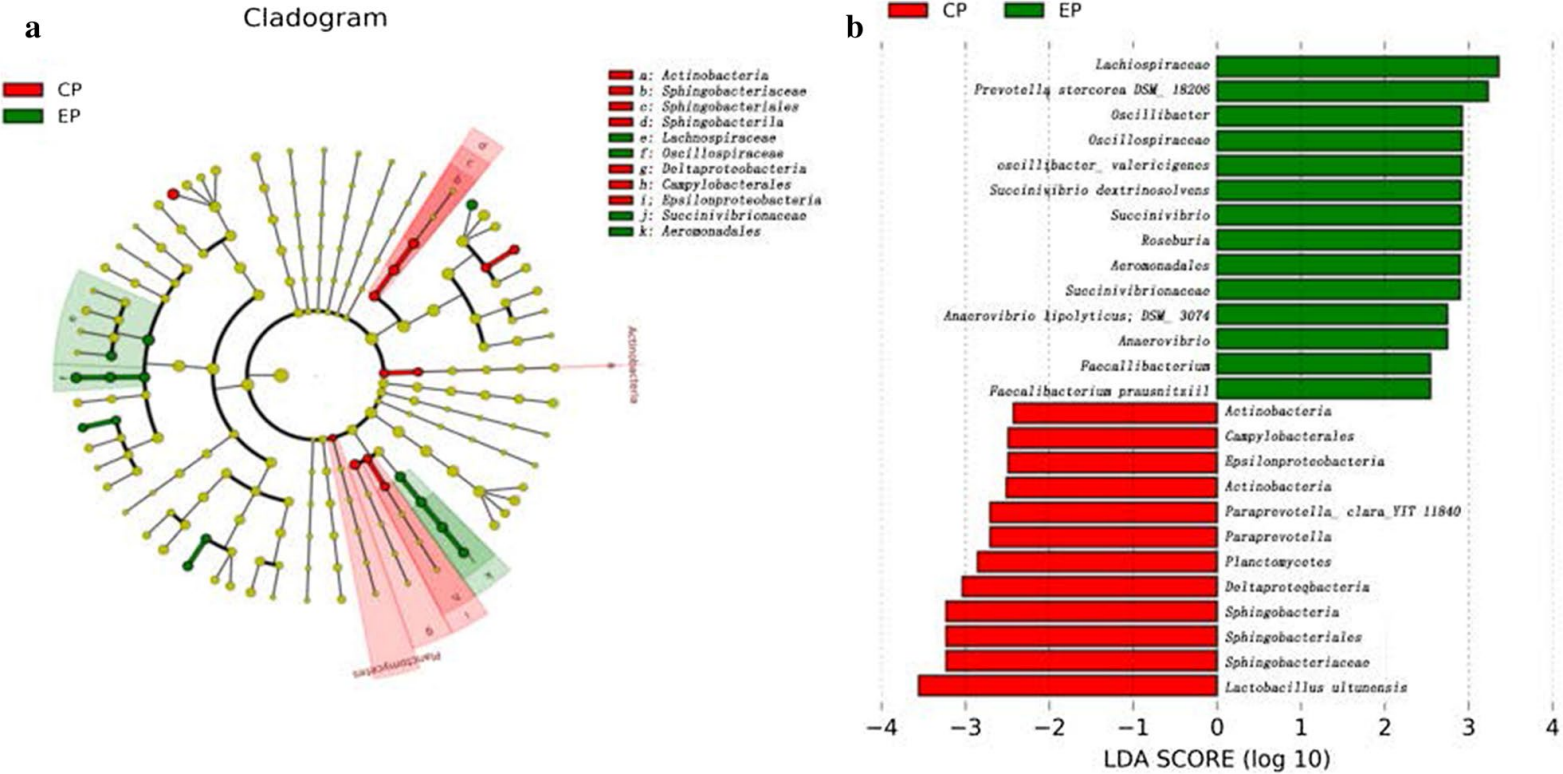
Differential bacterial taxa analysis of the intestinal microbiomes in CP and EP pigs. **a** OTUs differentially represented at the genus level between EP and CP Chuying black pigs identified by LEfSe. Histogram showing the OTUs that were more abundant in the EP (green color) or CP (red color) Chuying black pigs, ranked by effect size. **b** Phylogenetic tree of the microbial communities in the CP and EP groups. The phylogenetic tree with taxonomic nodes, where the diameter of the nodes indicates relative abundance, shows the intestinal microflora in the CP and EP groups. Different groups are labeled with different colors. The red areas indicate that the bacterial species were more abundant in the CP group, and the green areas indicate that the bacterial species were more abundant in the EP group

LEfSe was performed to identify specific taxa that consistently varied in abundance between the CP and EP groups across the different locations and could thus be used as biomarkers. Based on a logarithmic LDA score of 2.0 as the cutoff, a total of 26 genera were significantly differentially represented in the two groups, with 12 genera more abundant in CP pigs and 14 genera more abundant in EP pigs (Fig. 3a). A cladogram of family- and genus-level abundance is shown in Fig. 3b. The genera *Campylobacter* and *Paraprevotella*, which are known to include potential pathogens of poultry and/or humans, were potential biomarkers in the CP group, while *Prevotella* was a potential biomarker in the EP group.

### Differences in the functional potentials of the intestinal bacterial communities in the CP and EP groups

**Fig. 4:**
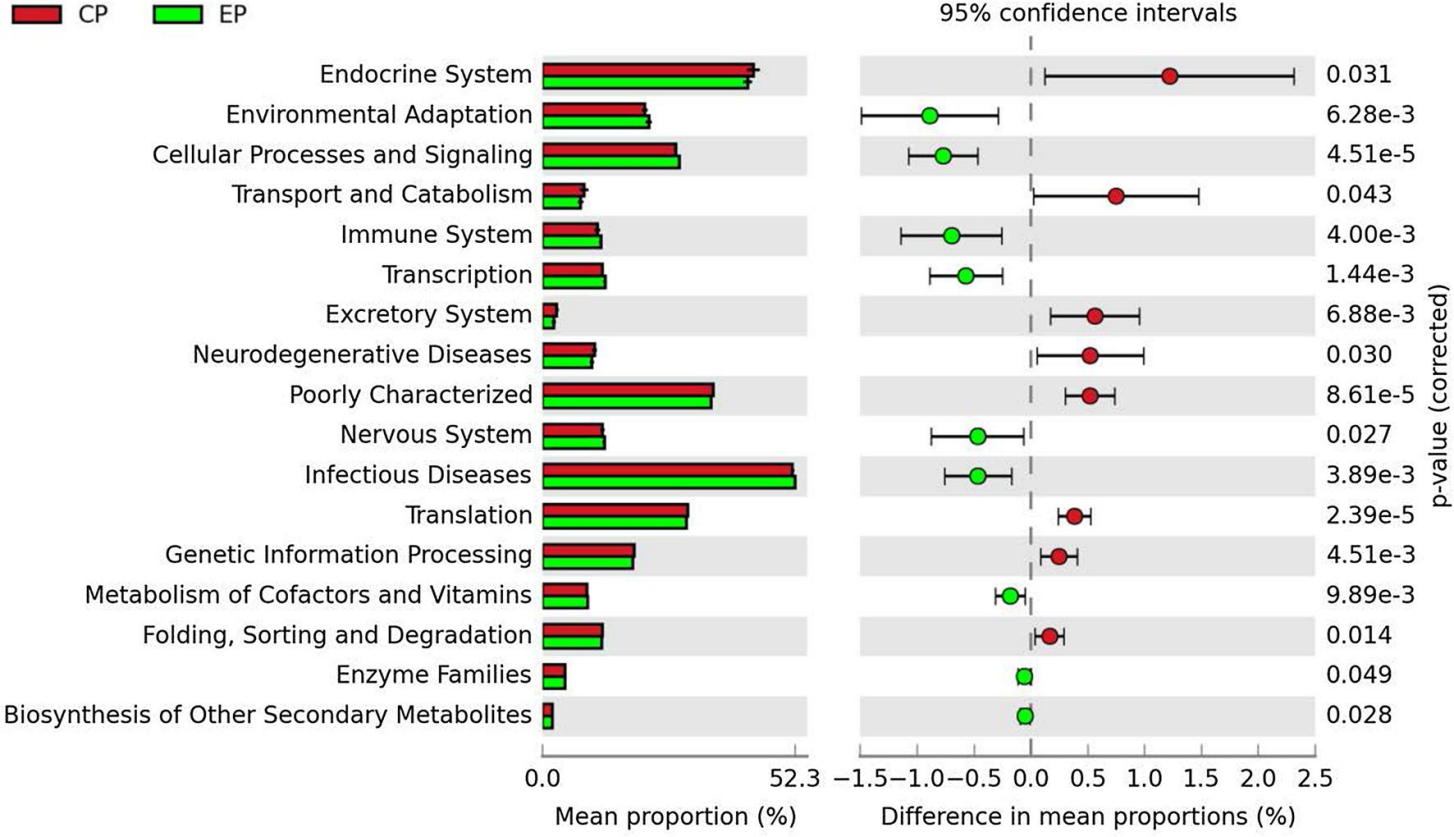
Predicted functions of the microbial flora in the CP and EP pigs. The third-level KEGG pathways are shown in the post hoc plot. The significance of gene distribution between groups was determined by ANOVA, with a P value < 0.05

To further predict how microbial flora differences potentially contribute to the differences in host phenotypes, PICRUSt (Langille et al. 2013) was used to analyze the microbial flora in the CP and EP groups. The statistical significance of gene distribution between the groups was determined using ANOVA. Many bacterial genes that are potentially related to growth and fat accumulation (such as transport and catabolism, translation, genetic information processing, endocrine system and amino acid folding, sorting, and degradation) were predicted to be enriched in the microbial flora of CP pigs (Fig. 4). On the other hand, bacterial genes potentially involved in disease resistance (such as environmental adaptation, the immune system, the nervous system, and metabolism of cofactors and vitamins) were predicted to be enriched in EP pigs (Fig. 4).

### Verification of the function of the differential flora at the cellular level

**Fig. 5:**
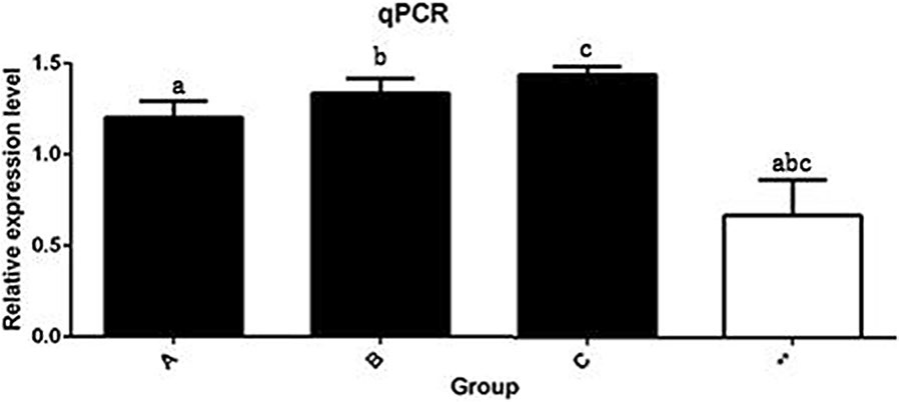
Bar chart of the qPCR results. **a** *Coprococcus* was treated with a concentration of 1 × 10^6^, **b** *Coprococcus* was treated with a concentration of 1 × 10^7^, **c** *Coprococcus* was treated with A concentration of 1 × 10^8^, ** represents the blank treatment control group

*Coprococcus* is a relatively common bacterium, and in this study, *Coprococcus* was identified as a key bacterium that potentially affected disease resistance in pigs. *Coprococcus* was selected for functional exploration in this study. After 12 h of intestinal epithelial cell treatment with *Coprococcus*, qPCR was used to identify SLA-DRB gene expression in the experimental group and the control group. The results showed that the concentrations of *Coprococcus* in the 1 × 10^6^, 1 × 10^7^, and 1 × 10^8^ solutions enhanced the expression of the SLA-DRB gene, making the expression level of the SLA-DRB gene in the experimental group significantly higher than that in the control group (*P < 0.05*). With increasing *Coprococcus* concentrations, its enhancing effect on SLA-DRB gene expression gradually increased (Fig. 5).

## Discussion

In this study, by investigating and comparing the characteristics and diversity of intestinal bacterial communities in CP and EP Chuying black pigs, our objective was to identify the different intestinal flora related to pig performance in pigs reared under different feeding patterns.

Disease is an important factor that restricts the development of the modern pig industry. Therefore, modern breeding techniques to improve disease resistance in animals through selective genetic breeding as well as appropriate inspection and quarantine measures have become important. Modifying intestinal flora species has become another important means of enhancing disease resistance. In this study, the abundances of *Lachnospiraceae* and *Roseburia*, which are related to environmental adaptation, the immune system, and antigen processing and presentation, were significantly higher in the EP group than in the CP group (*P* < 0.05). On the basis of functional prediction, we concluded that the EP group was superior to the CP group in disease resistance. The possible reason for this result is that EP pigs are more likely to be infected by pathogenic bacteria than CP pigs due to increased contact with the external natural environment. Therefore, EP pigs have an increased abundance of bacteria related to disease resistance in the intestinal tract, as the composition of their intestinal flora has adjusted to enhance their ability to adapt to the external environment. In addition, appropriate exercise can effectively enhance the physique of pigs. EP pigs can perform a wider range of activities than CP pigs, and the average amount of activity in EP pigs is greater than that in CP pigs; therefore, the composition of the intestinal flora is relatively healthy. The presence of *Lachnospiraceae* and *Roseburia* in the intestines can improve disease resistance in pigs (Fouhse et al. 2016), and changes in the microbial structure can also impact disease resistance in pigs (Roselli et al. 2017). Interestingly, research conducted by Relun et al. (2015) showed the relationships among traditional feeding methods, infectious diseases, and disease resistance and revealed that traditional feeding methods were conducive to enhancing disease resistance in pigs, but traditional pig rearing methods also increased the risk of infection. The results of the above studies were all consistent with those of this study, which to some extent suggests the credibility of the results of this study. Therefore, we suggest appropriately increasing contact between pigs and the outside environment during captivity and increasing the amount of exercise to improve disease resistance in pigs.

The physical health of animals is important, and the mental health of animals is also important. It has become an inevitable trend in the development of the modern breeding industry to ensure the mental health of animals and improve animal welfare. In this study, we found that *Paraprevotella*, *Lachnospiraceae*, and *Ruminococcaceae* were significantly more abundant in the intestines in the CP group than in those in the EP group (*P* < 0.05). Similarly, Jiang et al. (2015) found that the above microbes were present in lower concentrations in the intestines of depressed rats. The current results of the prediction of the function of different flora showed that the CP group had flora that has been associated with human neurological and neurodegenerative diseases, such as Alzheimer’s disease. In contrast, microflora related to the nervous system and cellular processes and signaling were found in the EP group. We can infer that a wide range of breeding environments may be more suitable for the development of animal mental health. In this study, pigs in the EP group had a larger exercise space, cleaner air, and less restricted activity than those in the CP group. In contrast, the CP pigs had narrow living spaces, a non-stimulating atmosphere, exposure to strong smells, and little contact with the external environment. As a result, captive pigs often develop neurological symptoms. At present, many scientific research teams have hypothesized that captive pigs suffer from mental depression, but related research is rare. The present study confirmed this hypothesis to a certain extent on the basis of the functional prediction of differential intestinal flora. Moreover, a large body of research on the brain-gut-microbiota axis confirms that gut microbes can indeed influence an animal’s mood and behavior by regulating the nervous system (Sampson and Mazmanian 2015). Another study also showed that gut microbes could produce neurotransmitters found in the human brain. Accumulating evidence supports the view that gut microbes influence central neurochemistry and behavior. Translational studies indicate that certain bacteria may have an impact on stress responses and cognitive function. Currently, it is possible to treat depression and neurological diseases by regulating the intestinal flora and thereby changing brain function (Dinan et al. 2017b). In addition, some research on the relationship between animal welfare and economic performance shows that good animal welfare has a positive impact on economic performance (Henningsen et al. 2018). Therefore, providing adequate outdoor exercise time for pigs may be beneficial to animal welfare to some extent, but how much outdoor exercise time is most effective in improving animal welfare deserves further study.

On the premise of ensuring animal health and welfare, improving the growth rate of pigs has become an important direction in the process of genetic breeding. The results of this study showed that the abundance of *Sphingobacteria*, *Deltaproteobacteria*, and *Lactobacillus*, which are related to intestinal fat accumulation, in the CP group were significantly higher than those in the EP group (*P* < 0.05). Moreover, the predicted intestinal flora function results showed that the intestinal functions related to xenobiotic biodegradation and metabolism, folding, sorting and degradation and other traits in the CP group were significantly enriched compared to those in the EP group (*P* < 0.05). The reasons for the above results may be that pigs in the CP group exercised less, were less disturbed by external factors, and were reared in an unchanging environment, leading to faster fat accumulation. Another research team conducted a similar study on the relationship between the composition of intestinal microorganisms and growth rate and fat accumulation. The results showed that the presence of *Sphingobacteria* and *Deltaproteobacteria* in the intestinal tract enhanced fat accumulation in the animals to some extent (Yang et al. 2016; Zhao et al. 2015). This result is consistent with our findings.

Finally, the results of this study showed that the abundance of *Aeromonadales* in the intestinal tract in the EP group was significantly higher than that in the intestinal tract in the CP group (*P* < 0.05). This may also be the cause of fat accumulation and slow growth in the EP group. The functional prediction results in the EP group also showed that the diversity of intestinal flora associated with infectious diseases was significantly greater than that in the CP group (*P* < 0.05). Thus, although free-range breeding guarantees improved animal welfare and increases disease resistance in pigs themselves, the probability of pigs being infected with pathogenic bacteria also increases due to increased contact with the external environment. Some members of the team studied the relationship between wild animals and the spread of disease and came to similar conclusions (Carrasco-Garcia et al. 2016). Therefore, we suggest improving epidemic prevention methods in free-range pig breeding systems; these methods will be conducive to the healthy growth of pigs and thus will have economic benefits.

In conclusion, this study found that bacteria associated with disease resistance and nervous system improvement were more abundant in the intestinal tracts of EP pigs than in in the intestinal tracts of CP pigs. However, microflora associated with fat deposition and mental illness were highly abundant in CP pigs. These findings may indicate poorer disease resistance, a lower incidence of mental disturbance, a higher rate of infection and a slower rate of growth in EP pigs than in CP pigs. Although the EP increases the risk of disease, it improves animal welfare and is more in line with the nature of animals than the CP. This study also elucidated the effect of *Coprococcus* on SLA-DRB gene expression in pig intestinal epithelial cells, providing reference data for studies on the influence of flora on host disease resistance. These findings can enhance our understanding of the relationship between intestinal flora and the rearing mode of pigs and provide a theoretical basis for subsequent studies on the regulation of host disease resistance by intestinal microflora. However, there are still some limitations of this study, as the findings based on 16S rRNA gene sequencing are somewhat limited. Therefore, in future studies, metagenomic sequencing and metabonomics should be used to conduct association analyses, and phenotypic determination methods should be employed to further explore the experimental results.

## Supplementary Information

**Table S1. ** Statistics of the paired-end reads in both groups.

**Table S2. ** Genus-level intestinal microbiota difference analysis results for the different groups.

## Data Availability

The raw sequencing data in this study were deposited in the NCBI Sequence Read Archive (SRA) under accession number SUB5539274.

## Abbreviations

CP: Complete intensive feeding pattern
EP: Extensive feeding pattern
PRRS: Porcine reproductive and respiratory syndrome
IACUC: Institutional Animal Care and Use Committee
BGI: Beijing Genomics Institute
OTUs: Operational taxonomic units
RDP: Ribosomal Database Project
PCA: Principal component analysis
ACE: Abundance-based coverage estimation
PCoA: Principal coordinate analysis
LefSe: Linear discriminant analysis effect size
rRNA: ribosomal RNA
KEGG: Kyoto Encyclopedia of Genes and Genomes
GO: Gene Ontology
